# Evaluation of Drought Tolerance of the Vietnamese Soybean Cultivars Provides Potential Resources for Soybean Production and Genetic Engineering

**DOI:** 10.1155/2014/809736

**Published:** 2014-04-07

**Authors:** Nguyen Binh Anh Thu, Quang Thien Nguyen, Xuan Lan Thi Hoang, Nguyen Phuong Thao, Lam-Son Phan Tran

**Affiliations:** ^1^School of Biotechnology, International University, Vietnam National University HCMC, Quarter 6, Linh Trung Ward, Thu Duc District, Ho Chi Minh City 70000, Vietnam; ^2^Signaling Pathway Research Unit, RIKEN Center for Sustainable Resource Science, 1-7-22 Suehiro-cho, Tsurumi, Yokohama 230-0045, Japan

## Abstract

Drought is one of the greatest constraints to soybean production in many countries, including Vietnam. Although a wide variety of the newly produced cultivars have been produced recently in Vietnam through classical breeding to cope with water shortage, little knowledge of their molecular and physiological responses to drought has been discovered. This study was conducted to quickly evaluate drought tolerance of thirteen local soybean cultivars for selection of the best drought-tolerant cultivars for further field test. Differences in drought tolerance of cultivars were assessed by root and shoot lengths, relative water content, and drought-tolerant index under both normal and drought conditions. Our data demonstrated that DT51 is the strongest drought-tolerant genotype among all the tested cultivars, while the highest drought-sensitive phenotype was observed with MTD720. Thus, DT51 could be subjected to further yield tests in the field prior to suggesting it for use in production. Due to their contrasting drought-tolerant phenotypes, DT51 and MTD720 provide excellent genetic resources for further studies underlying mechanisms regulating drought responses and gene discovery. Our results provide vital information to support the effort of molecular breeding and genetic engineering to improve drought tolerance of soybean.

## 1. Introduction

Soybean (*Glycine max *L. Merrill), primarily produced by the United States, Brazil, Argentina, China, and India, is currently considered as one of the most important oilseed crops all over the world [1]. Surprisingly, the world's biggest soybean consumers are the East Asian and Pacific countries, including China, Japan, Thailand, and Vietnam. Soybean's consumption as food products and animal feeding materials in Vietnam has radically grown in the last few years because of its widely recognized health-related benefits [2–4]. According to the statistics of 2012 from the United States Department of Agriculture, Vietnam produced approximately 270,000 tons of soybean with a total cultivated area of 180,000 hectares [5]. However, the local soybean supply only meets 18% nationwide demand due to low soybean productivity that predominantly resulted from abiotic stresses of which drought is the major constraint [6]. This is also a great challenge for the plant productivity many countries are facing, especially in arid areas where water resource is more restricted [7–10]. Therefore, gaining a better understanding of the mechanisms regulating plant adaptation to drought for maintaining plant growth, development, and productivity in water deficit regions is an important goal of many plant biologists and breeders worldwide [11]. In case of soybean production, drought severely affects soybean growth and development and may cause yield loss by approximately 40% in the worst year [10, 12, 13]. Each year, Vietnam still has to import 2.5 million tons of soybean [6]. As a result, development of soybean elite cultivars, which can sufficiently cope with water scarcity, has been an important task for soybean research community in Vietnam [14]. Thanks to soybean breeder's efforts, many soybean hybrid cultivars with improved productivity under drought have been recently developed by different research institutions and applied across the country [6].

A number of assessment methods have been exploited to quickly examine drought tolerance ability of soybean cultivars under stressed and nonstressed conditions based on their root and shoot growth rates [15]. It is well established that root length is one of the primary traits that support plants to tolerate the limited water conditions [16]. Thus, analyzing dynamics of root growth under severe drought conditions is important to specify the contribution of roots to drought adaptation [17]. In soybean, roots are distributed in the top soil when water is sufficient, but under water deficit, extensive root growth and development occurs deeper in the soil profile [17, 18]. Early establishment of the root system (seedling vigor) could be one of the important traits in the selection of soybean genotypes for improvement of soybean production in drought-prone areas [12]. Shoot growth rate of soybean is reduced by drought during vegetative growth and early reproductive development. However, soybean plants with strong drought-tolerant ability can be recovered after rewatering for certain days [19]. It has been reported that studies on plant stress physiology not only provide valuable information for agricultural practices and water-saving control but also enable identification of contrasting cultivars used in screening for candidate genes for development of improved drought-tolerant crops by genetic engineering [12, 13, 20–22]. Until recently, little research has been undertaken to examine phenotypic differences concerning drought tolerance among Vietnamese soybean cultivars. In a recent study, Ha et al. [23] have assessed the water loss and the shoot and root growth rates of an improved drought-tolerant local soybean cultivar (DT2008) and the reference cultivar Williams 82 (W82) under normal and drought conditions, but this study was limited due to the small number of tested varieties [20].

In this study, thirteen local cultivated varieties and the reference W82 were assessed under normal and drought conditions to reveal their morphological and physiological variations in response to water shortage. The objectives were to determine quickly which cultivar(s) possesses the best drought tolerance which might be suggested to be used in soybean production and to identify the most drought-sensitive and the most drought-tolerant cultivars from investigated phenotypes for further screening for differentially expressed drought-responsive candidate genes by expression analysis. Our results suggest that DT51 is the highest drought-tolerant cultivar, while MTD720 is the lowest one among all the cultivars examined. Thus, DT51 can be recommended to be used in farm production in the country, and these two contrasting cultivars, DT51 and MTD720, can be subjected to further differential studies to gain an insight into regulatory mechanisms of drought response and to identify useful genes for engineering soybean plants.

## 2. Materials and Methods

### 2.1. Plant Materials

In this study, 13 Vietnamese soybean cultivars collected from Can Tho University (MTD176, MTD720, MTD751, MTD765, MTD772, MTD775-2, and MTD777-2) and Vietnam Legumes Research and Development Center (DT20, DT22, DT26, DT51, DT84, and DT96) were used along with the reference phenotype W82.

### 2.2. Net House Conditions and Cultivation Techniques

All plants in the present study were cultivated inside a net house that helped to maintain a consistent temperature range (28–30°C) and a relative humidity (60–70%), together with a photoperiod of 12 h light and 12 h dark conditions. Initially, one seed was sown at 2 cm depth in each plastic tube with parameters specified below which was filled with a premixed standard potting soil. Irrigation was thoroughly undertaken every single day to ensure the distribution of identical water amount for individual plant.

### 2.3. Examination of Root and Shoot Growth at Seedling and V3 Stages under Well-Watered Conditions

Two screening methods using two different tube systems described in [24] were applied to examine physical growth of plants at certain stages under well-watered conditions. For seedling stage assessment, 30 plastic tubes (40 cm in height and 6.5 cm in diameter) were adhered to a tray representing each cultivar. After 12 days of planting, each tube was cut longitudinally in order to safely isolate the whole root system from potting soil. On the other hand, the V3-stage assessment (21 days after sowing) was implemented with also 30 plastic tubes (80 cm in height, 10 cm in upper diameter, and 6.5 cm in bottom diameter)/cultivar.

### 2.4. Drought-Induced Treatments

Sixty 4-day-old seedlings/cultivar grown in plastic tube system (80 cm in height and 10 cm in diameter), which have relatively the same height, were selected for drought-induced treatment. Regular irrigation was discontinued after 12 days of planting to initiate the 15-day-drought treatment. Soil moisture contents (SMC) were monitored at 5-day intervals (*n* = 3) using moisture balance (Shimadzu, Japan). For control, another set of plants was maintained from each variety under well-watered conditions. After 27 days of planting, the whole root systems from both drought-treated and well-watered groups were gently removed from soil for measurement of physical lengths and dry matter (DM).

### 2.5. Assessment Methods

Taproot and shoot lengths of each plant (*n* = 30) were measured immediately after its removal from soil. For determination of root and shoot dry matters (*n* = 30), the whole root and shoot systems were kept in drying oven at 65°C for 24 h before being weighed using an analytical balance (Satorius, Germany). Relative water content (RWC) of 27-day-old plants treated with drought was measured as described in [23]. The aerial parts of plants (*n* = 15) developed under both well-watered and drought conditions were measured to determine the sample fresh weight (FW). Subsequently, fully turgid weights (TW) of all the samples were determined after being soaked in deionized water overnight and gently wiped with absorbent paper to avoid extra moisture. The immersion process was undertaken under room light and temperature. Finally, the plants were dehydrated at 65°C for 48 h to measure dry weight (DW). RWC was calculated as

(1) $$\begin{array}{c} \text{RWC}\left(\%\right)=\left[\frac{\left(\text{FW}-\text{DW}\right)}{\left(\text{TW}-\text{DW}\right)}\right]\times 100. \end{array}$$

Drought-tolerant index (DTI) was calculated as described in [25]. Five seeds of each variety were geminated separately in each of the 5 plastic tubes (25 cm in height and 30 cm in diameter) (*n* = 25). The plants were maintained under well-watered conditions in net house. For drought treatment, water was withheld from 12-day-old plants for 15 days. The percentage of nonwithered plants was determined after 1, 3, 5, 7, 9, 11, 13, and 15 days after water withholding. After drought treatment, the plants were reirrigated for 15 days. The percentage of recovered plants was identified after 1, 3, 5, 7, 9, 11, 13, and 15 days of reirrigation. The drought-tolerant index of soybean varieties (referred to as a surface of a radar chart, comprised of multiple axes) was calculated as

(2) DTI=12sinα(D1R1+R1D3+D3R3+R3D5+D5R5    +R5D7+⋯+D15R15+R15D1),

where *D*_*n*_ is the percentage of nonwithered plants after *n* day(s) of drought treatment, *R*_*n*_ is the percentage of recovered plants after *n* day(s) of reirrigation, and *α* is the equal inner angle of the radar chart, which is formed by multiple axes (*D*_*n*_ and *R*_*n*_). In this case, *α* = 360/2*n* and the number of equal inner angles (2*n*) is 16.

### 2.6. Statistical Analysis

The data were analyzed using SAS (version 9.13, by SAS Institute, Inc., Cary, NC, USA). Differences among soybean cultivars in separated experiments were estimated with* Proc* GLM procedure. Duncan's test was subsequently applied to classify the cultivars into homogenous subgroups denoted by common letters. Mean values were shown on the figures, and error bars represent the standard errors.

## 3. Results and Discussion

### 3.1. Root and Shoot Lengths at Seedling and V3 Stages under Normal Growing Conditions

In crop plants, root growth is an important trait because of its essential role in water uptake. Stable and vigorous cultivars, which can produce their longer taproots to reach water source from deeper soil layer, would be considered as candidates that might have better tolerance to water deficit than those with shorter taproots [16]. Therefore, the root features were used to assess drought tolerance ability of the 13 local soybean cultivars, whereas the shoot-related traits were used as reference criteria in our evaluation.

**Figure 1:**
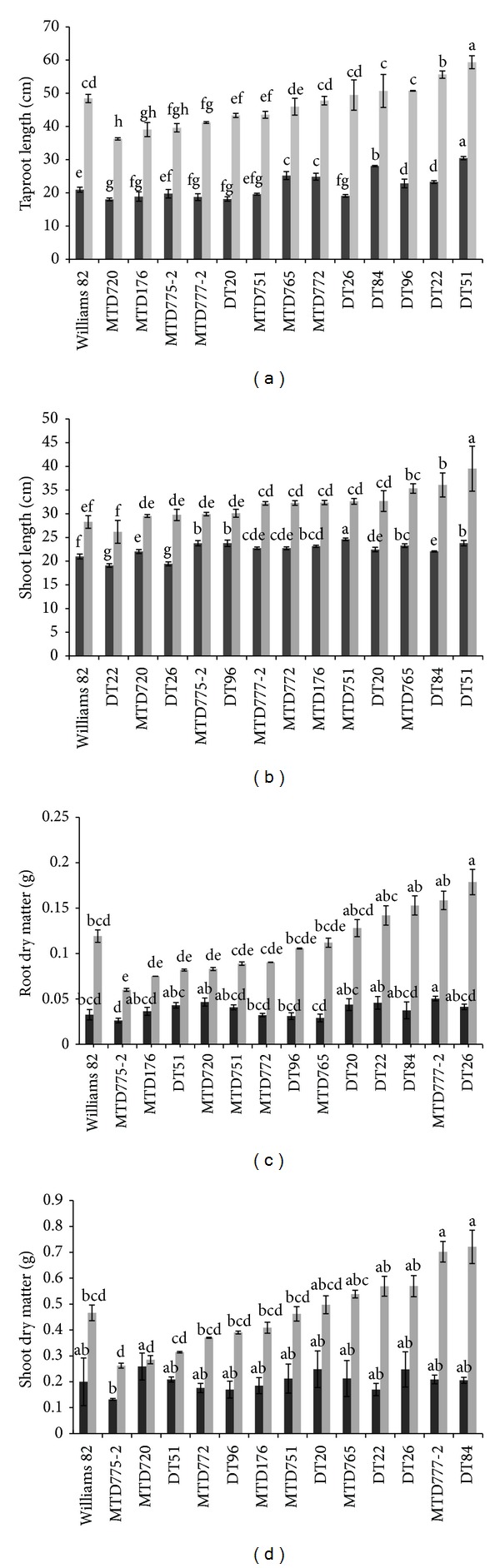
The root and shoot developments at 12-day-old seedling stage (black bars) and V3 stage (grey bars) of 13 soybean cultivars and the reference cultivar W82 under normal conditions. Roots and shoots were collected individually for measurement of the length and dry matter (DM) at day 12 after sowing and at V3 stage. (a) Tap root length. (b) Shoot length. (c) Root DM. (d) Shoot DM. Error bars represent standard error (*n* = 30). Different letters indicate significant difference at each developmental stage according to Duncan's test (*P* < 0.05 level).

The tube system was applied to compare the root and shoot traits among soybean cultivars in early developmental stage under normal growing conditions. After 12 days of seedling stage, significant difference for taproot length was detected (Figure 1(a)). On the basis of the taproot length data, 14 cultivars were classified into three groups. Four cultivars, W82, MTD775-2, MTD751, and DT26, fell into the medium taproot category (length 19–22 cm). MTD176, MTD777-2, DT20, and MTD720 were classified as short taproot length cultivars (length <19 cm), whereas DT51, DT84, MTD765, MTD772, DT22, and DT96 were classified as long taproot length members (length >22 cm). Among all the soybean cultivars examined, DT51 possessed the longest taproot length (30.5 cm) and MTD720 showed the shortest taproot length (18 cm), suggesting that DT51 might have the highest tolerance capacity, while MTD720 might have the lowest tolerance capacity to drought. We also observed a significant difference in shoot length of the examined cultivars at seedling stage (Figure 1(b)). On the basis of their shoot length, the tested cultivars could be divided into 3 groups: high (>23.5 cm), medium (22–23.5 cm), and short (<22 cm) groups. Five cultivars, including DT84, MTD720, W82, DT26, and DT22, were found to belong to the short shoot length category. DT20, MTD772, MTD777-2, MTD176, and MTD765 were classified as medium shoot length cultivars, whereas MTD751, DT51, DT96, and MTD775-2 were classified as high shoot length cultivars.

During examination of root characteristics at V3 stage under well-watered conditions, we found that there was a significant difference in taproot length among the cultivars (Figure 1(a)). According to the results, DT51, DT22, DT96, and DT84 were classified into the long taproot group (>50 cm). DT26, W82, MTD772, MTD765, MTD751, DT20, and MTD777-2 had medium taproot length (40–50 cm). The remaining varieties, including MTD775-2, MTD176, and MTD720, showed short taproot length (<40 cm). Among 14 varieties, DT51 exhibited the longest taproot length (59.3 cm), while MTD720 had the shortest root length (36.2 cm). DT51 also had the highest shoot length, making it a member of the high shoot length group (>35 cm) that also includes DT84 and MTD765. A number of cultivars, such as DT20, MTD751, MTD176, MTD772, and DT96, exhibited medium shoot length (30–35 cm), while MTD720, MTD775-2, DT26, DT22, and W82 fell into the low shoot length category (<30 cm) (Figure 1(b)).

These data together demonstrated that DT51, which had the longest root length and high shoot length, and MTD720, which displayed the shortest root length and short shoot length, at both seedling and V3 stages, might be the two contrasting drought-responsive cultivars.

### 3.2. Root and Shoot DM at Seedling and V3 Stages under Normal Growing Conditions

With regard to root DM, our data indicated that there was a significant difference in root DM among the cultivars at both seedling and V3 stages (Figure 1(c)). At seedling stage, high root DM group included MTD777-2, MTD720, and DT22 (>0.045 g). DT20, DT51, DT26, MTD751, DT84, and MTD176 had medium root DM (from 0.035 to 0.045 g), while W82, MTD772, DT96, MTD765, and MTD775-2 showed low root DM (<0.035 g). At V3 stage, the high root DM group included DT26, MTD777-2, and DT84 (>0.15 g). DT22, DT20, W82, MTD765, DT96, and MTD772 exhibited medium root DM (0.09–0.15 g), whereas MTD751, MTD720, DT51, MTD176, and MTD775-2 had low root DM (< 0.09 g). There was a slight difference in shoot DM among all the cultivars at seedling stage. MTD720, DT20, DT26, MTD765, and MTD751 had higher shoot DM (>0.2 g) than others, such as those belonging to medium shoot DM group, including DT51, MTD777-2, DT84, W82, and MTD176 (0.18–0.2 g), and those classified into low shoot DM group, including MTD772, DT96, DT22, and MTD775-2 (<0.18 g). Significant differences were recorded at V3 stage (Figure 1(d)). All cultivars could be divided into high (>0.5 g), medium (0.4–0.5 g), and low (<0.4 g) groups. DT84, MTD777-2, DT26, DT22, and MTD765 had high shoot DM, whereas medium group included DT20, W82, MTD751, and MTD176. The remaining cultivars (DT96, MTD772, DT51, MTD720, and MTD772) displayed low value of shoot DM.

These data suggested that the differences in taproot and shoot lengths are not well correlated with root and shoot DM. A similar result was observed in previous study of Manavalan et al. [24]. This might be explained by the fact that, although a decrease of total DM may be due to growing conditions, the distribution of biomass may also result from change in resource pools, leaf senescence, the reduction in photosynthesis and cell division, and the change in cell wall composition [16, 26, 27].

### 3.3. RWC under Normal and Drought Conditions

**Figure 2:**
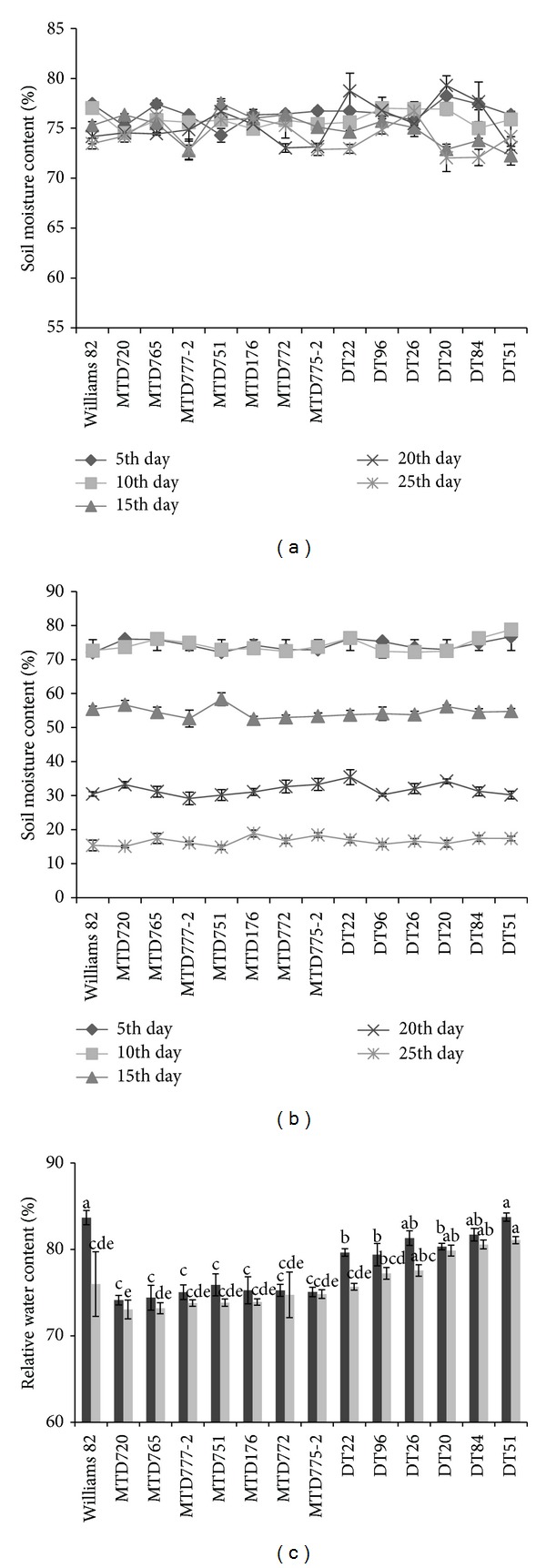
Examination of RWC of 13 soybean cultivars and the reference cultivar W82. For drought treatment, water withholding was applied to 12-day-old plants for 15 days. SMC was recorded in each pot of each cultivar at 5-day intervals during the measurement of RWC of the soybean cultivars. (a) SMC was measured under well-watered condition. (b) SMC was measured under drought condition. Error bars represent standard error (*n* = 3). (c) RWC under normal (black bars) and drought conditions (grey bars). Error bars represent standard error (*n* = 15). Different letters indicate significant difference within a treatment according to Duncan's test (*P* < 0.05 level).

Evaluation of RWC of various plants, especially under drought, will provide information about their tolerance levels in response to stress conditions [28]. This value highlights potential cultivars with better tolerance and thus higher yield, which exhibit higher RWC under drought. Thus, to further examine the contrasting drought responsive phenotypes of DT51 and MTD720, we determined the RWC of these two cultivars together with other local soybean cultivars and W82 during both normal and drought conditions. The SMC was monitored during drought treatment to ensure the similar SMC levels among different pots (Figures 2(a) and 2(b)). As a result, under both normal and drought conditions, DT51 showed the highest RWC (83.74% and 81.07%, resp.), and MTD720 displayed the lowest RWC (74.14% and 73.07%, resp.) (Figure 2(c)). These results suggested that DT51 and MTD720 are the highest and lowest drought-tolerant cultivars, respectively.

### 3.4. DTI under Normal and Drought Conditions

**Figure 3:**
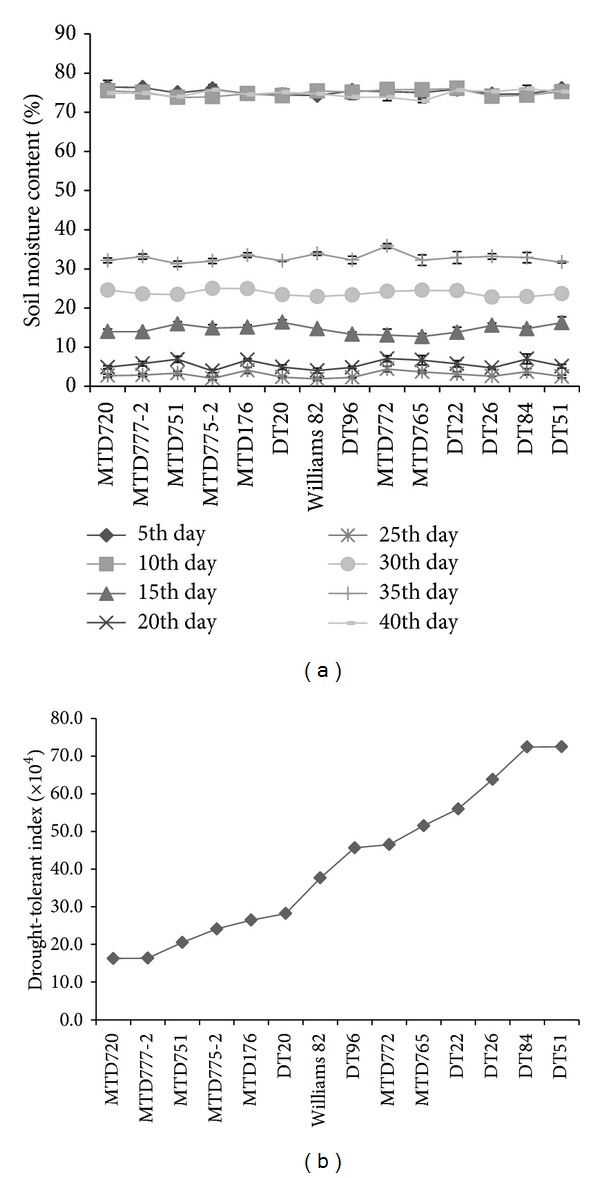
Examination of DTI after drought treatment for 13 soybean cultivars and the reference cultivar W82. (a) For the assessment of the DTI in soybean cultivars, the SMC was measured at 5-day intervals from germination to reirrigation with drought duration of 15 days. Error bars represent standard error (*n* = 3). (b) DTI values were determined by the percentage of nonwithered and recovered plants after 1, 3, 5, 7, 9, 11, 13, and 15 days of the drought exposure and reirrigation (*n* = 25/cultivar).

As a means to evaluate more exactly drought-tolerant capacity of the tested 13 soybean cultivars, we examined the DTI that represents survival and recovery rates of plants after drought treatment. This method was shown to be useful and time-saving by [25] in evaluating the drought-tolerant capacity in rice. Mau et al. (2010) also performed this method to compare the drought tolerance of several Vietnamese soybean varieties [29]. In our experimental pipeline, we performed a drought treatment in the tube system to trigger early withered state in soybean plants. The SMC was recorded every 5 days during the drought treatment and reirrigation periods (Figure 3(a)). As a result, MTD720 showed the lowest DTI (16.29 × 10^4^), whereas DT51 displayed the highest DTI (72.52 × 10^4^) (Figure 3(b)). These results firmly support that DT51 and MTD720 are the two cultivars with the most contrasting drought-responsive phenotypes. Thus, DT51 was identified as the highest drought-tolerant cultivar, whereas MTD20 was identified as the highest drought-sensitive cultivar.

### 3.5. Root and Shoot Growths under Normal and Drought Conditions

**Figure 4:**
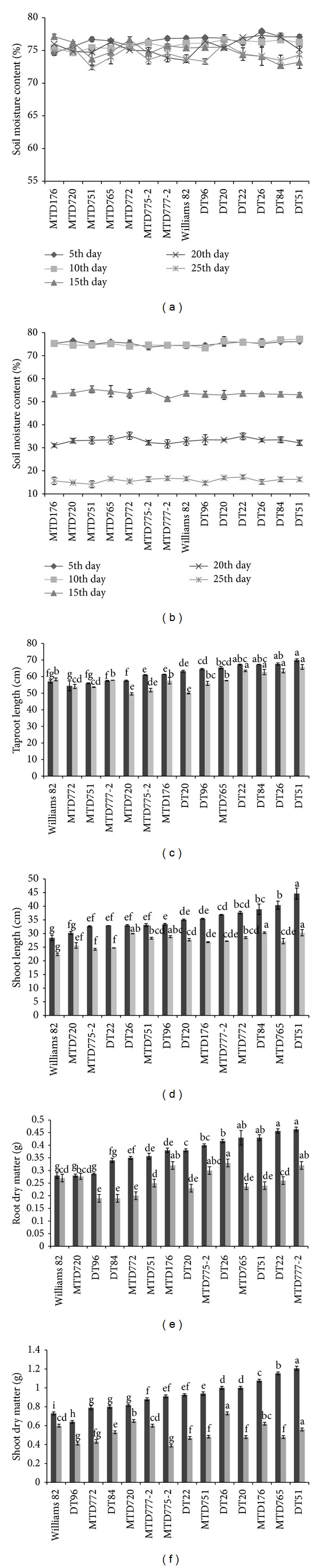
The root and shoot developments under normal (black bars) and drought (grey bars) conditions of 13 soybean cultivars and the reference cultivar W82. For drought treatment, water withholding was applied to 12-day-old plants for 15 days. SMC was recorded in each pot of each cultivar at 5-day intervals. (a) SMC was measured under well-watered condition. (b) SMC was measured under drought condition. Error bars represent standard error (*n* = 3). (c) Tap root length. (d) Shoot length. (e) Root DM. (f) Shoot DM. Error bars represent standard error (*n* = 30). Different letters indicate significant difference within a treatment according to Duncan's test (*P* < 0.05 level).

To examine morphological and physiological differences in response to water shortage of the two contrasting drought-responsive cultivars, DT51 and MTD720, we performed a drought treatment and evaluated root and shoot growths of all the tested cultivars. Previously, Read and Barlett reported that both shoot and root lengths of soybean were decreased under water deficit conditions [30]. Moreover, root and shoot DMs are decreased under low water availability in soil [31]. We observed similar tendency in this study as all 13 local soybean cultivars showed decreases in both shoot and root growths at different levels after a period of 15 days of drought treatment using the tube system. The height of the tube was 80 cm, which was suitable for development of taproot during the drought treatment. The SMC for each cultivar was monitored periodically during the experiment as shown in Figures 4(a) and 4(b).

**Figure 5:**
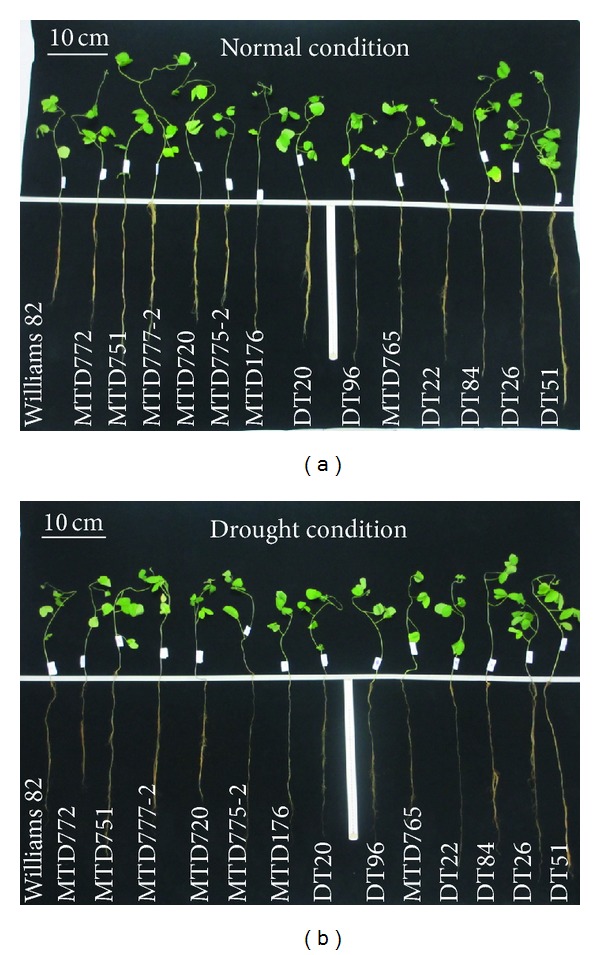
Examination of morphology for 13 soybean cultivars and the reference cultivar W82. (a) Morphology of 27-day-old plants grown under normal condition. (b) Morphology of 27-day-old plants grown under drought condition.

After 27 days of sowing, DT51 displayed the highest taproot length under both conditions (69.95 cm and 65.82 cm), whereas MTD720 exhibited the shortest taproot length under drought (49.5 cm) (Figure 4(c)). We observed that the shoot length was more significantly inhibited than the root length by stress (Figures 4(d) and 5), which was also supported by a previous study [30]. DT51 exhibited the highest shoot length under both normal and drought conditions (44.6 and 30.3 cm, resp.) (Figure 4(d)), and interestingly also had the highest decrease of shoot length during stress when compared with its respective one obtained under normal conditions. It is important to note that, in plants, the inhibition of shoot length was a primary response to water deficit, which might extend the period of soil water availability and plant survival as an adaptive response [32]. On the other hand, MTD720 exhibited short shoot length under both normal and drought conditions (30.22 and 25.66 cm, resp.) (Figure 4(d)).

In addition, we also investigated the effects of drought on root and shoot DMs. We found that all of the cultivars showed decrease in root and shoot DMs under stress (Figures 4(e) and 4(f)). However, again, we did not observe a clear correlation between the root length and the root DM, as well as the shoot length and the shoot DM, suggesting that the DM data might not be used as an important feature for evaluation of drought tolerance. Published literature also suggests that plant biomass should not be regarded as a sensitive parameter, because the decrease in biomass accumulation is mainly affected by long-term stress conditions [33].

Taken together, we recorded DT51 and MTD720 as two cultivars having contrasting drought-tolerant features, of which DT51 was the highest drought-tolerant cultivar, whereas MTD720 was the lowest drought-tolerant cultivar. This finding was also supported by a differential expression analysis of a subset of* GmNAC* genes [34], which are known as transcriptional factors involved in regulation of plant response to drought [35–37]. The expression of drought-responsive* GmNAC*s in roots of DT51 and MTD720 was significantly different. The better drought-tolerant capacity of DT51 was shown to be related to the higher number of drought-inducible* GmNAC* genes, as well as the higher number of* GmNAC* genes with higher transcript accumulation in comparison with MTD720 [34].

## 4. Conclusions

In this study, we have examined the shoot and root growths, as well as RWC and DTI of 13 local soybean cultivars and the reference W82 at different stages under well-watered and water deficit conditions. Our data suggested that, among the 14 tested varieties, DT51 and MTD720 could be considered as the highest drought-tolerant and drought-sensitive varieties, respectively. These two cultivars could be used as contrasting genetic resources for determination of drought-responsive genes with differential expression, which are potentially involved in regulation of drought responses in soybean, and mutations responsible for drought tolerance, enabling us to understand drought tolerance mechanisms in soybean. Additionally, the differentially expressed genes may serve as promising candidates for genetic engineering of soybean with the aim of improving soybean productivity under adverse environmental conditions. On the basis of our data, DT51 can be subjected to further intensive field tests prior to subjecting it to the production chain.
